# The toxicology of orally administered Δ^9^-tetrahydrocannabinol in sheep

**DOI:** 10.1016/j.toxrep.2026.102221

**Published:** 2026-02-11

**Authors:** Sarah A. Stevens, Scott H. Edwards, Glenys K. Noble, Colin J. Scrivener, Christopher E. Petzel, Christopher D. May, Zi Xuan Tai, Bronwyn L. Blake, Kenneth C. Dods, Leon N. Warne, Gaye L. Krebs

**Affiliations:** a School of Agricultural, Environmental and Veterinary Sciences, Charles Sturt University, Wagga Wagga, NSW 2650, Australia; b ChemCentre, Bentley, WA 6983, Australia; c SAGE Consultancy, Dianella, WA 6059, Australia; d The Vet Pharmacist, East Fremantle, WA 6158, Australia; e Curtin Medical School, Curtin Health Innovation Research Institute, Curtin University, Bentley, WA 6002, Australia

**Keywords:** Δ^9^-tetrahydrocannabinol, Body weight, Feed intake, Industrial hemp, Ruminants, Troponin I

## Abstract

**Introduction:**

As part of a larger study to assess the potential value of industrial hemp biomass (the low Δ^9^-tetrahydrocannabinol (Δ^9^-THC) variety of *Cannabis sativa* L.) as a feed for ruminants, a pharmacokinetics study of Δ^9^-THC, one of the main cannabinoids of interest, was undertaken. In undertaking this study, the toxicology of orally administered Δ^9^-THC was incidentally investigated and was discussed in the current paper.

**Methods:**

Eight Merino ewes were administered with two *per os* (PO) doses of 88.5 mg Δ^9^-THC/kg bodyweight (BW) 12 h apart. Blood samples were taken periodically to determine the pharmacokinetics of Δ^9^-THC but also to check blood troponin I concentrations post dose administration as in the pharmacokinetics study there were adverse effects to the heart following intravenous administration of Δ^9^-THC.

**Results:**

Following the PO dose of Δ^9^-THC, the pharmacokinetics were able to be determined (and were published separately); however, significant adverse clinical signs were observed in the sheep. Clinical signs included ataxia (stumbling, swaying), abnormal posture (head held low, head resting on the wall of the pen, back legs crossed over), somnolence, wool pulling, increased salivation, a prolonged reduction in feed intake and consequent decrease in BW. Plasma troponin I concentrations were elevated in three of the eight sheep following the PO dose of Δ^9^-THC.

**Conclusions:**

The adverse clinical signs noted have implications for iHemp biomass as a potential feed for ruminants due to both animal health and production effects. Further research is required to investigate appropriate feeding strategies for iHemp biomass to be utilised as a feed for ruminants.

## Highlights

• Δ^9^-THC administration caused ataxia, somnolence and abnormal posture in sheep.
• Wool pulling persisted for up to seven days post administration of Δ^9^-THC.
• Feed intake was decreased during the first week post administration of Δ^9^-THC.
• Troponin I concentrations were elevated in three sheep post Δ^9^-THC administration.

## 1 Introduction

For industrial hemp (iHemp) biomass, the low Δ^9^-tetrahydrocannabinol (Δ^9^-THC) variety of *Cannabis sativa* L., to be a suitable feed for ruminants it needs to be nutritionally suitable and not cause any adverse health or production effects. Toxicity following oral exposure to Δ^9^-THC has occurred in a range of species, including donkeys [1], monkeys [2], [3], dogs [3], [4], cats [5], rats and mice [6]. A range of clinical signs have been identified across species, including but not limited to; depression, lethargy, ataxia, tachycardia, bradycardia, tachypnoea, bradypnea, anorexia, hyperactivity to stimuli, muscle tremors, mydriatic pupils, hypersalivation, disorientation, hypothermia, constipation and urinary incontinence [1], [3], [4], [5], [6].

The maximum allowable concentration for total THC (total THC calculated based upon Δ^9^-THC + 0.877 * THCA) to be present in an iHemp crop in New South Wales (NSW), Australia is 1 % (Hemp Industry Act 2008). The effects upon ruminants grazing iHemp biomass with this concentration of THC is largely unknown. Following feeding sheep diets containing either 0.002 % total THC [7] or 0.015 % total THC [8] or 0.067 % total THC [9], [10], feeding goats diets containing 0.02 % total THC [11] and feeding cattle diets containing 0.065 % total THC [12], no significant adverse effects were noted; however, following feeding cattle diets containing 0.1 % total THC some adverse effects were identified [13]. These adverse effects included a significant decrease in respiratory and heart rates, along with changes in behaviour including increased yawning, salivation, nasal secretion, somnolence, pronounced tongue play, unsteady gait, unusually long periods of standing, abnormal posture and a reduction in feed intake [13]. Furthermore, in a case study where five cattle ingested a high Δ^9^-THC hemp hay (exact concentration unknown) clinical signs of Δ^9^-THC toxicosis included muscle tremors, mydriasis, hypersalivation, reluctance to move, incoordination and frothing from the mouth, with ultimately fatalities occurring in four of the five animals [14]. Following IV administration of either 160 mg Δ^9^-THC/kg BW or 18.7 mg Δ^9^-THC/kg BW to two different sheep [15], a range of clinical signs were observed before fatality occurred in both sheep, attributed to a cardiac effect of Δ^9^-THC, of which was identified by plasma troponin I assay ( [16]; unpub data). Whether feeding sheep iHemp biomass with cannabinoids closer to the maximum allowable limit in Australia will cause adverse effects is largely unknown, necessitating further research before it should be fed to ruminants.

In undertaking a study of the pharmacokinetics of Δ^9^-THC in sheep (results on the pharmacokinetics of Δ^9^-THC published separately by Stevens et al. [17]), it became apparent that *per os* (PO) administration resulted in adverse effects in sheep, and these effects were the focus of the current study.

## 2 Materials and methods

The study was conducted at the Department of Primary Industry and Regional Development (DPIRD) Animal Nutrition Unit at Wagga Wagga, NSW. Approval was obtained from both CSU Animal Ethics Committee (Protocol Number: A22440) and NSW DPIRD Animal Ethics Committee (Protocol Number: OAEC-0547) prior to commencement of the study. The study was compliant with the Animal Research Act 1985 (as amended) in accordance with the Australian Code of Practice for the Care and Use of Animals for Scientific Purposes.

### 2.1 Experimental design

The experimental protocol for the larger pharmacokinetics study that the current study was part of has been previously reported [17]. Results reported in the current study are only of the clinical signs that arose as part of the pharmacokinetics study. In summary, eight Merino ewes of approximately 24 months of age were administered with two PO doses of 88.5 mg Δ^9^-THC/kg BW 12 h apart. This dosage was based on the worst-case grazing scenario, in which sheep would graze an iHemp crop at the maximum allowable limit, being 1 % in NSW (Hemp Industry Act 2008). The dosage took into account an approximate feed intake of 3.5 % BW [8] and the potential for all the tetrahydrocannabinolic acid (THCA) in the plant to be converted into Δ^9^-THC. For the final dose, a 50 % dose reduction was applied to reduce the potential of the dosage resulting in fatality.

All sheep were dosed according to BW. The required amount of a 73.2 % Δ^9^-THC resin (Little Green Pharma®, Western Australia) was weighed using calibrated scales (Sartorius, Germany), then dissolved in dimethyl sulfoxide (DMSO) (Sigma-Aldrich, USA). All doses were administrated via an orogastric tube, followed by 10 mL of DMSO to flush the tube to ensure full delivery of the dose solution. A new orogastric tube was used for each sheep to ensure there was no cross contamination.

The experimental period comprised a total of 62 d, including a dietary and housing adaptation period of 34 d followed by a 28 d sample collection period.

### 2.2 Clinical observation

The sheep were closely observed, and monitoring sheets were filled out for each sheep based upon clinical signs reported by Wagner et al. [13]. Clinical observations recorded included physiological (changes in pupil size, heart rate, respiratory rate, capillary refill, salivation, nasal secretion, reflexes) and behavioural (changes in gait, time standing/sitting, posture, somnolence, response to stimuli, wool pulling).

### 2.3 Feed intake and analysis

Throughout the experimental period the sheep were fed *ad libitum* a ration comprised of wheaten chaff and a commercially available sheep pellet. Refusals were measured daily to enable determination of daily dry matter intake (DMI).

Before analysis, all DMI data were assessed for the model assumptions of normality by histograms and plotting residuals. Dry matter intake data were analysed using Repeated Measures Analysis, using the Mixed Model procedure in SAS statistical program (SAS Institute Inc., 1997). For the analysis, “week” was examined as the fixed effect with “animal” examined as a random effect and initial liveweight examined as a covariate. By referring to the Bayesian Information Criterion, the most appropriate covariance structure for each analysis was determined [18]. The final model included the fixed effect of “week”, with “animal” as a random effect. All data were recorded as least squares means ± SE of the least square means. Differences were significant when *p* < 0.05.

### 2.4 Blood collection and analysis

Blood samples were taken from a catheter located in the jugular vein for each sheep. Prior to dosing a 0 h blood sample was taken. Following PO delivery of the first half of the dose, blood samples were taken at 12 h, 24 h, 36 h, 48 h, 72 h, 96 h, 120 h, 144 h, 168 h, 192 h, 216 h, 240 h, 264 h, 288 h, 312 h, 336 h, 504 h and 672 h for subsequent analysis of cannabinoids as part of the larger pharmacokinetics study but also for troponin I concentrations.

Troponin I was analysed at Gribbles Veterinary Pathology, Clayton Victoria using the Siemens Centaur instrument with a Chemiluminescent Immunoassay method. Troponin I concentrations were analysed to check whether there was an effect upon the heart following Δ^9^-THC administration, as occurred previously in humans [19], [20] and also when undertaking the pharmacokinetics of Δ^9^-THC in sheep ( [16]; unpub data). Troponin I concentrations were measured at the timepoint when plasma Δ^9^-THC concentrations were at their greatest in each animal (range 12 – 168 h post dosing)*.* As the assay utilised in the current study was not specific to sheep, there was no reference range provided for normal plasma troponin I concentrations. In the literature, a normal reference range for plasma troponin I concentrations in healthy sheep varies; however, a range of 0–100 ng/L can be assumed based upon the available literature [21], [22], [23] and all time zero samples associated with the larger pharmacokinetics study analysed previously by the authors.

## 3 Results

### 3.1 Clinical signs

Within 24 h of administration of the dose all eight sheep developed ataxia (stumbling, swaying), abnormal posture (head held low, head resting on the wall of the pen, back legs crossed over), somnolence and wool pulling to varying degrees, with two sheep also exhibiting increased salivation. Clinical signs became less apparent within 24–48 h; however, wool pulling persisted for 7 d for all sheep.

### 3.2 Feed intake

**Fig. 1:**
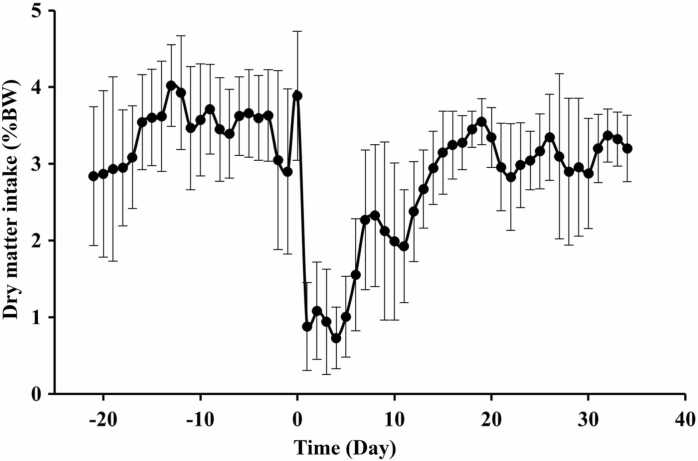
Average ± SD dry matter intake over 62 d for eight Merino ewes PO administered with 177 mg Δ^9^-THC/kg BW.

For the first 12 h following PO administration of Δ^9^-THC, there was a transient increase in DMI followed by a significant (*p* < 0.001) anorexic effect over the first week post dosing (Table 1), with DMI not returning to (almost) pre-dosing levels until three weeks post dosing (Fig. 1). As a consequence of the reduced DMI, there were changes in BW across the experimental period, from a mean ± SD BW of 47.75 ± 3.49 kg on Day 0 (day of administering the Δ^9^-THC), to 43.13 ± 3.40 kg on Day 14, and 49.50 ± 3.23 kg on Day 21 post Δ^9^-THC administration.

**Table 1:** Weekly average dry matter intake for eight Merino ewes PO administered with 177 mg Δ^9^-THC/kg BW.

**Dry matter intake (% BW)**^a^				
One week pre-dosing	First week post-dosing	Second week post dosing	Third week post dosing	p-value
3.4 (±0.138)^a^	1.2 (±0.138)^c^	2.3 (±0.138)^b^	3.3 % (±0.138)^a^	< 0.0001

a Values are least squares means ± standard error of the least squares means. Values within a row with different superscripts are significantly different (*p* < 0.05).

### 3.3 Plasma troponin I concentrations

Mean ± SD plasma troponin I concentration was 96.3 ± 87.57 ng/L (range 13 – 275 ng/L), with three of the eight sheep having plasma troponin I concentrations > 100 ng/L (144, 146 and 275 ng/L).

## 4 Discussion

Δ^9^-THC toxicity is multisystemic resulting in various physiological and behavioural changes [24], with some variation between species. The sheep developed ataxia, abnormal posture, increased salivation, increased somnolence, and became anorexic which resulted in a concurrent reduction in BW. Similar clinical signs have been reported in other species, including cattle [13], [14], donkeys [1], monkeys [2], [3], dogs [3], [4], cats [5], rats and mice [6]. The timing of the first appearance of clinical signs varied between animals but clinical signs were evident in all sheep within the first 24 h post administration. Following ingestion of cannabis buds two donkeys exhibited clinical signs 24–36 h post ingestion and made a full recovery within 24 h following the appearance of clinical signs [1]. Similarly in dogs, clinical signs post ingestion appeared anywhere from 5 min to 96 h with clinical signs lasting between 30 min to 96 h post first appearance [4], [25]. The timeline of the appearance and disappearance of clinical signs in other species generally aligned with that observed for sheep in the current study, except for the wool pulling and anorexia.

### 4.1 Wool pulling

The primary cause of the wool pulling identified in the current study was unknown. Intake of Δ^9^-THC may have potentially induced a side effect similar to delusional infestation which occasionally occurs in humans who use marijuana. Delusional infestation is a feeling of the skin being infested with organisms or inanimate particles [26], which can result in itching, biting and crawling sensations in/on the skin [27]. As wool pulling occurred until at least the maximum concentration of Δ^9^-THC was reached in the plasma, which occurred at mean ± SD 97.50 ± 65.55 h post dose administration [17], a similar condition as delusional infestation may have contributed to the sheep wool pulling. Alternatively, wool pulling in sheep can occur due to the stress of an indoor environment or overcrowding [28]; however, these sheep were well-adapted into their indoor individual pens where they were housed for a total of 69 d, with wool pulling only evident during the first week post dosing and at no other time. Additionally, wool pulling may occur due to nutritional needs not being met, such as when sheep have a deficiency in sulphur [29], zinc [30] or fibre [31]. As the sheep were eating inconsistently post dose administration, there was the potential that the nutritional state of the sheep may have contributed to the observed wool pulling and assist in explaining why wool pulling ceased once the sheep returned to their normal (pre-dose) eating pattern.

### 4.2 Feed intake

PO administration of 87.5 mg Δ^9^-THC/kg BW (half ultimate dose) resulted in a short-term (< 12 h) increase in feed intake, which was followed by a profound anorexic effect over the first week post dosing and resulted in a short-term decrease in BW. A similar pattern was identified following IV administration of the l-isomer of Δ^9^-THC to sheep, with feed intake increased during the first 30 min post exposure (this effect was not observed following dosing with the d-isomer), before feed intake decreased 24 h post dosing of both the L- and d-isomer of Δ^9^-THC [32]. When iHemp biomass containing diets were fed to sheep there was no change in feed intake [7], [8]; however, when made into silage and fed to cattle there was a significant reduction in feed intake [13]. The difference between these feeding studies was most likely attributed to the cannabinoid concentration of the feed. The iHemp hay diet fed to the sheep contained 0.03 % Δ^9^-THC, which equated to an average daily intake of 0.94 mg Δ^9^-THC/kg BW [8], whilst the cattle were fed iHemp silage containing 0.1 % Δ^9^-THC, which resulted in an average daily intake of 3.1 mg Δ^9^-THC/kg BW [13]. As Δ^9^-THC intake increases, effects upon feed intake appear to become more pronounced. Therefore, if iHemp biomass is to be used as a feed for ruminants the concentration of Δ^9^-THC will need to be carefully considered to ensure there are no adverse effects upon animal production.

### 4.3 Troponin I

Troponin I is a protein found in myocardial muscle that is released when damage occurs [33]. Elevation in plasma troponin concentrations have been identified in humans post cannabis use with troponin concentrations found to trend down after cessation of cannabis use [19], [20]. In the current study three of the eight sheep had increased plasma troponin I concentrations, indicating the PO dose administered elicited a mildly toxic myocardial effect. Troponin I concentrations were only measured at one timepoint for each animal in the current study and, therefore, there may have been more than just three sheep to experience elevated troponin I concentrations post dose administration that were not detected at the timepoints selected.

In humans that use cannabis, the risk of cardiac complications increases with the concentration of Δ^9^-THC present in the product [34] and the duration of use [35]; however, once cannabis use is ceased, the risk of cardiac complications decreases rapidly [34]. Based on the elevated plasma troponin I concentrations identified in the current study, if ruminants were to graze iHemp biomass there is the chance of an adverse myocardial effect occurring. The risk of an adverse myocardial effect occurring would potentially increase if ruminants were to graze iHemp for an extended period, with a concentration of Δ^9^-THC closer to the maximum allowable limit of 1 %. Therefore, further research is required to investigate what prolonged exposure of Δ^9^-THC, as would occur under grazing conditions, would mean for ruminant health before iHemp biomass should be incorporated into ruminant diets.

### 4.4 iHemp as a feed

To date iHemp biomass has mainly been investigated as part of a ruminant diet [7], [8], [9], [11], [13] and not the full diet. There is the potential for iHemp biomass to be utilised as only a proportion of the diet, as occurred in the aforementioned studies; however, it was still valid to investigate the worst-case scenario in which iHemp biomass comprised the majority of the ration, as there are anecdotal reports (from Australia and New Zealand) that it has been utilised as a grazing crop.

From the current study, administration of Δ^9^-THC at 177 mg/kg BW (equivalent to a 0.5 % iHemp crop), caused adverse clinical signs to arise; however, when a dose of 18.7 mg/kg BW (equivalent to an approximately 0.05 % Δ^9^-THC iHemp crop) was PO administered to a sheep, no adverse effects were identified [17]. Furthermore, no clinical effects were observed when sheep were fed diets containing either 56 % iHemp stubble with 0.002 % total THC for 56 d [7], or 42 % iHemp hay with 0.015 % total THC for 22 d [8] or 20 % spent hemp biomass with 0.067 % total THC for 56 d [9], [10]. Similarly, no clinical effects were observed following feeding goats iHemp biomass containing 0.02 % total THC for 70 d [11] or cattle fed spent hemp biomass containing 0.065 % total THC for 28 d [12]; however, cattle fed a hemp silage containing 0.1 % total THC for 6 d displayed a range of clinical signs [13]. Based upon these studies, somewhere between a 0.05 % and 0.1 % Δ^9^-THC crop is the upper safe limit for ruminants to graze iHemp biomass without compromising productivity or welfare. Further research is required to confirm what the upper safe limit may be.

It should be noted that the dose solutions utilised in the current study were comprised of a cannabinoid resin and effects from other plant secondary metabolites normally present in iHemp biomass, may influence the levels of Δ^9^-THC that ruminants can graze before an adverse effect is observed, with potential varietal differences [36]. Additionally, there are a range of other cannabinoids in iHemp biomass [37], which have the potential to exert a pharmacological effect upon the animal [38], [39] and contaminate animal products post consumption [7], [9], [40]. One of the other main cannabinoids of iHemp is cannabidiol (CBD). Generally, iHemp biomass contains a greater concentration of CBD than Δ^9^-THC [37], [41]; however, adverse pharmacological effects tend to be lessor for CBD than Δ^9^-THC but may still occur [42]. Sheep exposed to the same concentration of CBD as the concentration of Δ^9^-THC administered in the current study had no adverse effects identified post administration [15]. Further research into the other cannabinoids contained in iHemp biomass is warranted before iHemp or iHemp byproducts should be a feed for ruminants.

## 5 Conclusions

The concentration of the cannabinoids, especially Δ^9^-THC, in iHemp biomass will be an important aspect to consider if it is to be a feed for livestock to ensure there are no adverse effects upon the animal and consequently, production characteristics. Based off the current study and available literature, the upper safe limit for Δ^9^-THC consumption for ruminants would lie somewhere between a 0.05 % and a 0.1 % Δ^9^-THC iHemp crop, before a toxic effect was observed.

## CRediT authorship contribution statement

**Gaye L. Krebs:** Writing – review & editing, Supervision, Project administration, Methodology, Investigation, Formal analysis, Conceptualization. **Colin J. Scrivener:** Writing – review & editing, Supervision, Project administration, Methodology, Investigation, Formal analysis, Conceptualization. **Glenys K. Noble:** Writing – review & editing, Supervision, Project administration, Methodology, Investigation, Formal analysis, Conceptualization. **Scott H. Edwards:** Writing – review & editing, Supervision, Project administration, Methodology, Investigation, Formal analysis, Conceptualization. **Sarah A. Stevens:** Writing – review & editing, Writing – original draft, Supervision, Project administration, Methodology, Investigation, Formal analysis, Conceptualization. **Bronwyn L. Blake:** Methodology, Funding acquisition, Conceptualization. **Zi Xuan Tai:** Validation, Methodology, Investigation. **Christopher D. May:** Validation, Methodology, Investigation. **Christopher E. Petzel:** Writing – review & editing, Supervision, Project administration, Methodology, Investigation, Conceptualization. **Leon N. Warne:** Writing – review & editing, Resources. **Kenneth C. Dods:** Methodology, Funding acquisition, Conceptualization.

## Funding

This research was funded by 10.13039/501100009207AgriFutures Australia, grant number PRJ-013007 (Opening the gates to hemp-grazed livestock in Australia—Phase 2).

## Declaration of Competing Interest

The authors declare that they have no known competing financial interests or personal relationships that could have appeared to influence the work reported in this paper

## Data Availability

Data will be made available on request.
